# Preparation of Superhydrophobic Surface on Titanium Alloy via Micro-Milling, Anodic Oxidation and Fluorination

**DOI:** 10.3390/mi11030316

**Published:** 2020-03-17

**Authors:** Xiao Zhang, Yi Wan, Bing Ren, Hongwei Wang, Mingzhi Yu, Anqi Liu, Zhanqiang Liu

**Affiliations:** 1 Key Laboratory of High Efficiency and Clean Manufacturing, School of Mechanical Engineering, Shandong University, Jinan 250061, China; xiaozhang9591@163.com (X.Z.);; 2 National Demonstration Center for Experimental Mechanical Engineering Education, School of Mechanical Engineering, Shandong University, Jinan 250061, China; 3 Department of Mechanical and Aerospace Engineering, University of Florida, Gainesville, FL 32611, USA

**Keywords:** titanium, micro-milling, anodic oxidation, fluorination, micro/nanostructure, superhydrophobic surface

## Abstract

The superhydrophobic surface has a great advantage of self-cleaning, inhibiting bacterial adhesion, and enhancing anticoagulant properties in the field of biomedical materials. In this paper, a superhydrophobic surface was successfully prepared on titanium alloy via high-speed micro-milling, anodic oxidation and fluoroalkylsilane modification. The surface morphology was investigated by scanning electron microscope and a laser scanning microscope. The surface wettability was investigated through the sessile-drop method. Firstly, regular microgrooves were constructed by micro-milling. Then, nanotube arrays were fabricated by anodic oxidation. Afterwards, fluoroalkylsilane was used to self-assemble a monolayer on the surface with a composite micro/nanostructure. Compared to polished titanium samples, the modified samples exhibited superhydrophobic properties with the water contact angle (CA) of 153.7° and the contact angle hysteresis of 2.1°. The proposed method will provide a new idea for the construction of superhydrophobic titanium surgical instruments and implants in the future.

## 1. Introduction

At present, with the aging of population, the incidence of cardiovascular diseases, such as thrombosis and hemangiomas, is greatly increasing. However, traditional vascular dredge surgery is very expensive and time-consuming, and will cause damage for the organs of patients. With the development of advanced manufacturing technology and modern medical technology, the appearance of stent therapy method has provided new approaches and opportunities for the treatment of cardiovascular diseases [1].

Currently, titanium and its alloys are ideal biomedical metal materials for their features of light weight, low elastic modulus, high specific strength, good corrosion resistance and biocompatibility [2,3,4]. They have been widely used in the field of implant materials, such as artificial joints, dental implants, artificial heart valves and cardiovascular stents. As for vascular implants, it is essential that they have good blood compatibility in order to achieve good treatment effects. However, the titanium alloy itself does not have antibacterial properties.

When implanted in the human body, it is susceptible to infection by bacteria in the surrounding environment, which will lead to surgical failure. In addition, during the process of service, the cardiovascular stent will make contact with the blood and interact with the platelets, red blood cells and plasma proteins, resulting in changes in blood components, and even coagulation and thrombosis, which will do harm for patients’ health [5]. Due to the adhesion of bacteria and platelets, the infections and thrombus formation still remain great challenges for the success of blood contact implants during long-term service.

Superhydrophobic surfaces were the surfaces with a water contact angle (CA) above 150° and a contact angle hysteresis below 5°. Materials with superhydrophobic surface show great potential, and have been widely applied in the field of biomedical materials, especially for cardiovascular stent materials, due to their unique characteristics, such as self-cleaning, low adhesion and reduced viscosity resistance [6,7,8,9,10]. For instance, it has been reported that superhydrophobic surfaces could hinder the adhesion of bacteria to surface so as to reduce the risk of instrument and implant infections [11]. Moreover, compared to smooth surfaces, implants with superhydrophobic surfaces can reduce the adsorption of proteins and the adhesion and deformation of platelets, which is believed to improve blood compatibility and anti-coagulation performance [12,13]. Furthermore, it will also be easier to clean up the surgical instruments due to the superhydrophobic surfaces [14].

In this case, the construction of superhydrophobic surfaces is quite essential for the clinical application of cardiovascular implants and surgical instruments, and the construction of superhydrophobic surfaces has drawn great attention from scholars around the world. After implantation in the human body, the surface of the implant firstly interacts with human tissues. Therefore, it is very important to modify the surface of implants to improve their antibacterial performance, corrosion resistance and blood compatibility before clinical applications. It has been reported that the surface wettability is mainly affected by the surface morphology and chemical composition of the materials [15,16]. Studies [17,18,19] have proven that the micro/nanostructures on implant surfaces have great effects on regulating the surface wettability. It has been found that the surface of many animals and plants in nature is superhydrophobic, such as lotus leaves and rice foliage, water striders legs surface, and so on [20]. Neinhuis et al. [21] found that the micro/nano composite structure on the surface of lotus leaves could reduce the surface energy, which was the main reason for their superhydrophobic property. Studies have shown that the surface of rice leaves also has a similar nanoscale, needle-like composite structure [22]. Meanwhile, the surface of the water striders’ legs has microscale, needle-like bristles and nanoscale grooves [23]. Therefore, in order to achieve superhydrophobic properties, it is necessary to prepare micro/nano composite structures on the surface of the samples, and study their effect upon the surface wettability.

Many methods for the preparation of superhydrophobic surfaces have been reported, such as chemical deposition, oxidation, acid etching, the hydrothermal method, and so on [24,25,26,27]. McCarthy et al. used an etching method to construct a rough surface structure and covered the surface with a low surface energy substance, polytetrafluoroethylene, to generate the superhydrophobic surface [28]. Brevnov et al. deposited a polymer film on the surface of aluminum alloy using the method of plasma deposition to achieve a superhydrophobic state [29]. However, most of the surface treatment methods employed to change the surface morphology and roughness of titanium have usually been under a single scale. Moreover, it is difficult to get controllable and uniform micro/nanostructures using the above methods. Therefore, a new method to prepare regular micro/nano composite structures is expected. Appropriate micro/nanostructures and low surface free energy are the key factors for the preparation of superhydrophobic surfaces. At present, micro-milling technology has promising applications on preparation of controllable, microscale surface modification [30], which provides a strong support for the surface modification of titanium. Cai et al. manufactured microstructures on stainless steel using micro-milling, and studied the effect of microstructure on surface contact angle [31]. In addition, Chen et al. fabricated the superhydrophobic coatings on gelatin-based films via fluorination, and studied its characterization and cytotoxicity [32]. The results showed that the cytotoxicity of the surface after fluorination modification was acceptable.

In the present study, micro-milling and anodic oxidation were used to fabricate a composite micro/nanostructure with regular microgrooves and nanotubes on the surface of the titanium alloy. Then the surfaces were modified by the fluorination method in order to reduce the surface free energy. The effect of the modification on the surface morphology and wettability were investigated.

## 2. Materials and Methods

### 2.1. Preparation of Microgrooves via Micro-Milling

**Figure 1:**
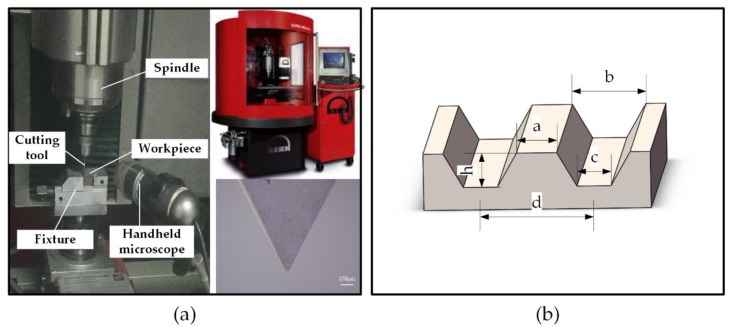
(**a**) Micro-milling machine tool and the milling cutter. (**b**) Geometrical dimensions of the microgrooves.

Ti-6Al-4V sheets with a dimension of 5 mm × 5 mm × 2 mm were polished by sandpapers from 400# to 2000# orderly. Then they were successively cleaned by ultrasonication in acetone, anhydrous ethanol and deionized water. The polished titanium samples were designated as P Ti. Microgrooves were constructed on the surface via micro-milling. The process of high-speed micro-milling was performed on a five-axis, high-precision micro machining center, using the V-shaped cutting tools with the blade angle of 60°, as shown in Figure 1a. The depth of the microgrooves h was set as 20 μm, and the space of two parallel adjacent microgrooves d was set as 200 μm, as shown in Figure 1b. The machining parameters of these experiments are listed in Table 1, which have been optimized through preliminary cutting experiments. The titanium samples processed via micromachining were designated as M Ti.

### 2.2. Construction of TiO_2_ Nanotubes via Anodic Oxidation

**Figure 2:**
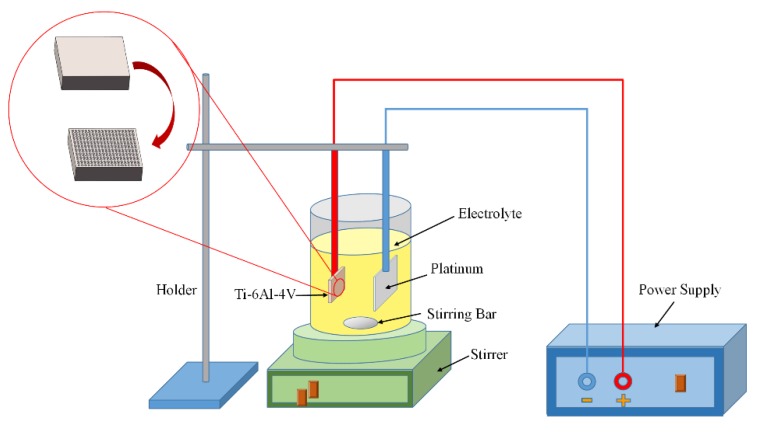
Diagram of anodic oxidation.

TiO_2_ nanotubes were constructed on the Ti-6Al-4V surface with microgrooves via the electrochemical method of anodic oxidation. The schematic of anodic oxidation was shown in Figure 2. Ti-6Al-4V sheets were linked to the anode of the power supply, while a platinum plate with a dimension of 10 mm × 10 mm × 0.1 mm was used as the cathode. The distance between the anode Ti-6Al-4V sheet and the cathode platinum plate was set as 40 mm. The electrolyte of ethylene glycol contained 0.25 wt% NH_4_F and 2.0 wt% deionized water, which was placed on a stirrer with stirring. The oxidation voltage was set as a constant voltage of 15 V. The anodic oxidation process was carried out at room temperature for 1 h. After that, the samples were softly rinsed with deionized water for further use. The resulting titanium samples were designated as MN Ti.

### 2.3. Fluorination on the Surface with Micro/Nanostructure

After the modification of micro-milling and anodic oxidation, the titanium specimens were immersed in a 1.0 wt% ethanol solution of 1H,1H,2H,2H-perfluorooctyltriethoxysilane (FAS-13, C_8_F_13_H_4_Si(OCH_2_CH_3_)_3_) at room temperature for 12 h. Then, the titanium specimens were heated and dried at 80 °C for 1h in a drying oven. The resulting titanium samples were designated as F Ti.

### 2.4. Specimen Characterization

The surface topographies of different titanium samples were characterized using scanning electron microscope (SEM, JSM-7610F, JEOL, Tokyo, Japan), and the chemical composition of the surfaces was tested by the energy-dispersive spectroscopy (EDS). The three-dimensional (3D) topographies of the samples were observed by a laser scanning microscope (LSM, VK-X200K, KEYENCE, Osaka, Japan).

### 2.5. Surface Wettability

The water contact angle and the contact angle hysteresis of the titanium alloy were measured using a contact angle goniometer (OCA15EC, Data-Physics, Filderstadt, Germany) by the sessile-drop method. The wetting agent was deionized water with a drop volume of 2 μL. No less than five measurements were conducted for every group of specimens.

## 3. Results and Discussion

### 3.1. Surface Morphologies

**Figure 3:**
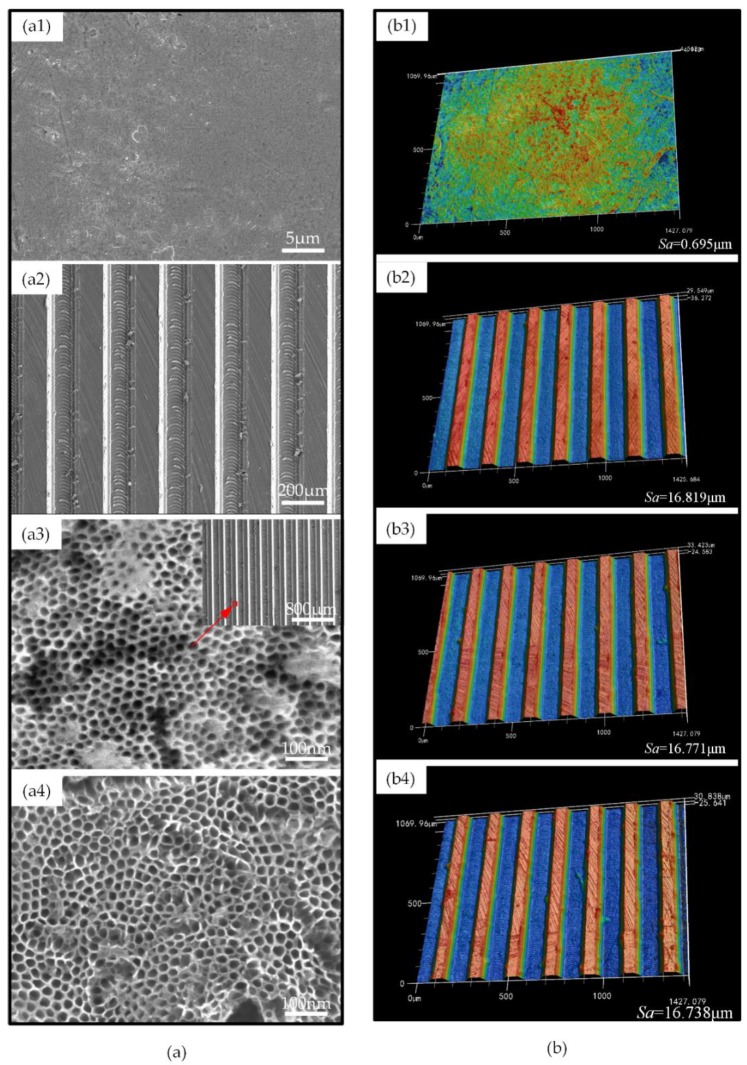
(**a**) Scanning electron microscope (SEM) images of (**a1**) P Ti, (**a2**) M Ti, (**a3**) MN Ti, (**a4**) F Ti. (**b**) laser scanning microscope (LSM) images of (**b1**) P Ti, (**b2**) M Ti, (**b3**) MN Ti, (**b4**) F Ti.

As shown in Figure 3a, the surface of P Ti was smooth, and the surface of M Ti showed high and low staggering peaks and valleys, with traces of the milling path at the bottom of the grooves. Moreover, there were few processing burrs on the M Ti surface. After anodic oxidation, closely arranged nanotubes were constructed on the surface of MN Ti. The inner diameter of the nanotubes was about 25–30 nm. Moreover, the nanostructure did not change the previous microstructures constructed by micro-milling. After fluorination, the surface morphology did not change a lot, compared to that of MN Ti. The 3D morphology of the surfaces characterized by LSM was shown in Figure 3b. From the results, it could be found that the surface of P Ti was smooth, with few scratches which were caused by polishing, and there was no obvious difference in the 3D shape of the surface of the M Ti, MN Ti, and F Ti sheets, which also indicated that any subsequent modification of anodic oxidation and fluorination did not change the microgroove structures.

As for the surface roughness, the P Ti had the smoothest surface with the Sa value of 0.695 μm. The surface roughness of M Ti significantly increased to 16.819 μm due to the existence of microgrooves.

After anodic oxidation and fluorination, the surface roughness did not change a lot, which also indicated negligible effects on the microstructures from the anodic oxidation and fluorination. The surface roughness results were consistent with the results of surface morphology.

### 3.2. Surface Chemical Composition

**Figure 4:**
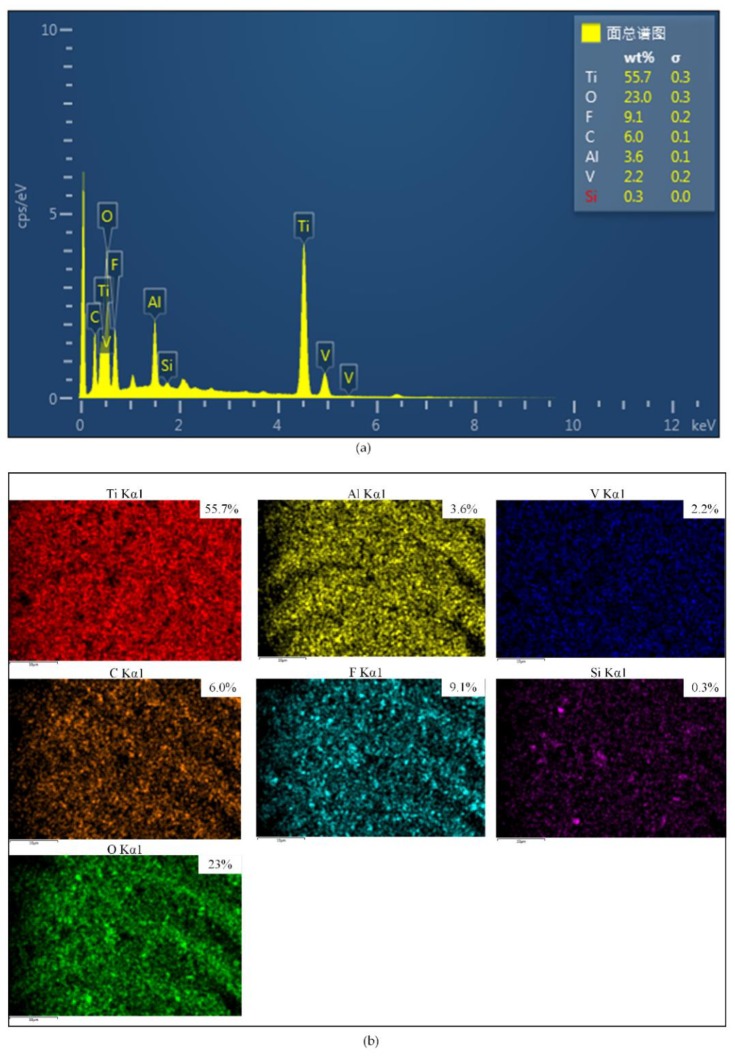
(**a**) Energy-dispersive spectroscopy (EDS) spectra of F Ti. (**b**) Surface chemical element distribution images of F Ti.

The chemical compositions and element distribution images of F Ti were shown in Figure 4. Besides the fact that the surface with fluorination consisted of the chemical elements F (9.1%), Si (0.3%) and C (6.0%), which indicated that a FAS film was successfully formed on the surface of F Ti. The FAS film could reduce the surface free energy. It has been demonstrated that the Ti-6Al-4V surface after anodic oxidation contained massive hydroxyl groups [33,34].

**Figure 5:**
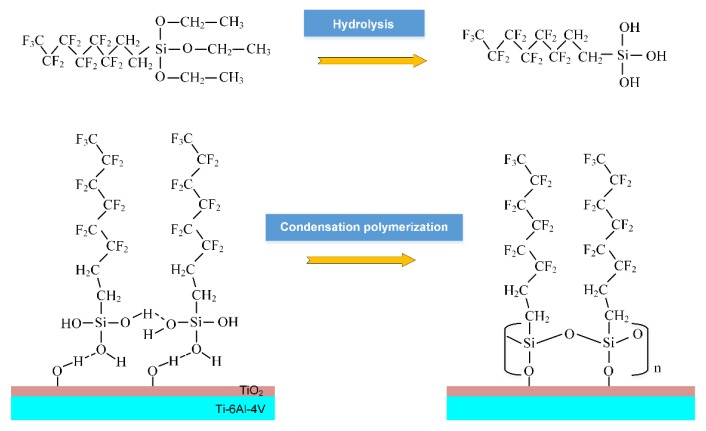
Schematic diagram of the chemical grafting process of fluorination.

As shown in Figure 5, the FAS molecules after the hydrolysis reaction were supposed to be strongly anchored onto the morphologically-modified Ti-6Al-4V substrate via covalent linkages, followed by condensation polymerization between the hydroxyl groups and silanol groups to form a self-assembled monolayer on the surface [35].

### 3.3. Surface Wettability

Wettability is a very important property of solid surfaces. The wettability of a solid surface is determined synergistically by the microscopic geometry and chemical composition of the surface. When the liquid drops on the solid surface, the liquid drops will contact with the substrate, forming a solid–liquid–gas, three-phase contact line. When the droplet reaches a steady state on the solid surface, the droplet will form a certain angle on the solid surface. At the intersection of the solid–liquid–gas three-phase contact, the angle between the tangent plane of liquid–gas boundary and the solid–liquid boundary is defined as the water contact angle of the droplet on the solid surface. The value of the contact angle is an important indicator for the qualitative analysis of the wettability of solid surfaces.

**Figure 6:**
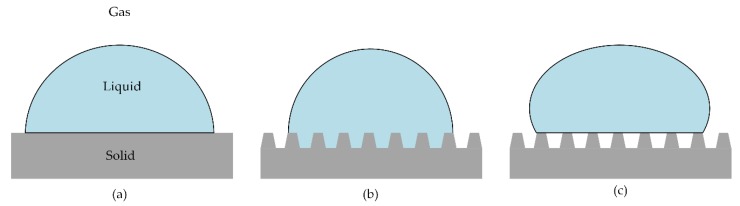
Schematic diagrams of different wetting status: (**a**) ideal status. (**b**) Wenzel status. (**c**) Cassie–Baxter status.

**Figure 7:**
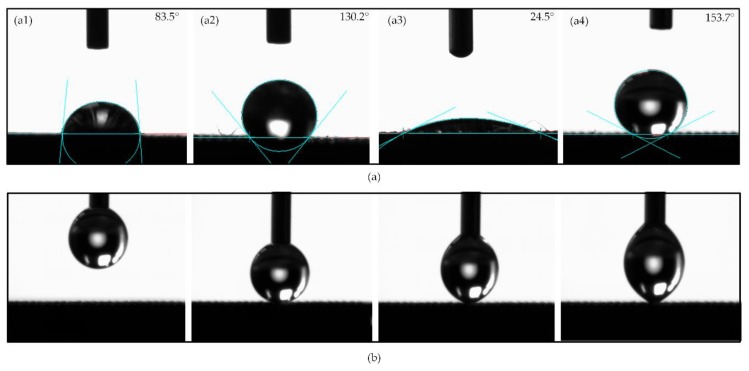
(**a**) Contact angle (CA) of (a1) P Ti. (a2) M Ti. (a3) MN Ti. (a4) F Ti. (**b**) The measurement process of CA of F Ti.

It has been widely reported that there are two main kinds of wetting status of droplets on a rough solid surface. They are the **Wenzel** state [36] and the **Cassie–Baxter** state [37], which are developed based on **Young’s** model [38] for smooth surfaces, as shown in Figure 6. The Wenzel theory describes that water droplets will fully infiltrate these structures and present a “pinning effect”. In contrast, according to Cassie–Baxter’s theory, CA will increase when there are microstructures on the surface, because air can be trapped in the gaps to form a layer of air cushion and support the water droplets. The results of the contact angle of different samples were shown in Figure 7a. In the paper, the contact angles were measured in the view direction along the microgrooves. The CA of the M Ti surface was 130.2°, which was hydrophobic. After anodic oxidation, the CA of the MN Ti surface was 24.5°, which turned to hydrophilic. After fluorination, the surface was converted from hydrophilic to superhydrophobic with the CA of 153.7°. Figure 7b showed the measurement process of the CA of the F Ti surface. Meanwhile, from the measurement process, it could also have been concluded that the water droplet was not easy to spread onto the F Ti surface

**Figure 8:**
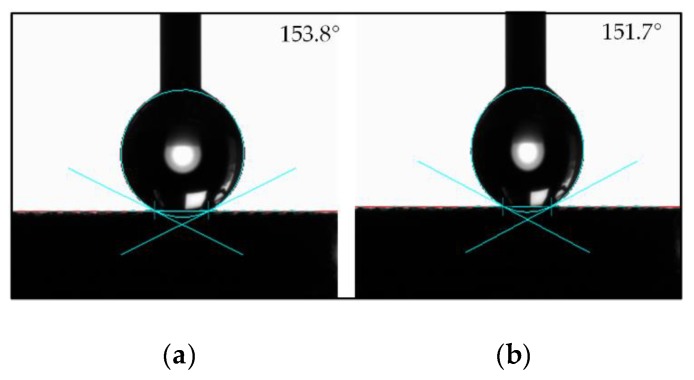
The contact angle hysteresis of F Ti: (**a**) Advancing contact angle θ_A_. (**b**) Receding contact angle θ_R_.

When the solid surface is hydrophobic to the droplet, the static contact angle alone is not sufficient to fully describe the wettability of the solid surface. Therefore, it is essential to measure the dynamic contact angle of a droplet on a solid surface. In order to characterize the dynamic contact angle of the surface reliably, the contact angle hysteresis of F Ti was measured using the method of the increase and decrease of droplet volume [39]. During the measurement process, as the volume of the droplet increased gradually, the contact angle also increased, and the contact boundary of the solid-liquid interface had a tendency to advance. When the volume of the droplet increased to a certain threshold, the contact boundary of the solid-liquid interface of the droplet would move outward, but keep still. The corresponding contact angle was defined as the advancing contact angle θ_A_. Conversely, as the volume of the droplet decreased gradually, the contact boundary of the solid-liquid interface tended to recede. When the volume of the droplet decreased to a certain threshold, the contact boundary of the solid-liquid interface of the droplet would move inward, but also keep still. The corresponding contact angle was defined as the receding contact angle θ_R_. The contact angle hysteresis was the difference value between the advancing contact angle and the receding contact angle. The smaller the contact angle hysteresis, the easier it is for the liquid to leave the surface. As shown in Figure 8, the contact angle hysteresis of F Ti was 2.1° (advancing contact angle θ_A_ = 153.8° and receding contact angle θ_R_ = 151.7°), which further revealed that the surface of F Ti was superhydrophobic.

The microstructure in this study showed high and low staggering peaks and valleys. Therefore, the structures might be easy to trap air when water droplets contact with the surface, so that the surface wetting state might be similar to the Cassie–Baxter state. CA of the polished surface (83.5°) can be considered as $\theta_{Y}$. The CA ($\theta_{W}$) of Wenzel state is as follows:

(1) $$\cos\theta_{W}=r\times \cos\theta_{Y}$$

where r is defined as the ratio of the real surface area to the projected surface area [40]. Obviously, r is always ≥ 1.

The CA ($\theta_{C}$) of the Wenzel state is as follows:

(2) $$\cos\theta_{c}=-1+\left(-1+cos\theta_{Y}\right)f$$

where f is defined as the ratio of the real surface area to the projected surface area. Obviously, f is always ≤ 1.

The geometrical dimensions of the microgrooves (Figure 1b) were designed as follows: the depth of the microgrooves h, width of the bulge a (120 μm), width of the upper side of the groove b and the width of the below side of the groove c. Thus, the contact angle under the Wenzel state and the Cassie–Baxter state were, respectively, as follows:

(3) $$\cos\theta_{W}=\frac{a+c+4h/\surd 3}{a+b}\cos\theta_{Y}$$

(4) $$\cos\theta_{C}=-1+\left(-1+cos\theta_{Y}\right)\frac{a}{a+b}$$

The calculated value of the CA of the M Ti surface under the Wenzel state was 84.2°, while the calculated value of the CA of the M Ti surface under the Cassie–Baxter state was 123.7°. The actual measured value was close to the theoretical calculated value. Therefore, the results showed that the contact angle state of the M Ti surface was close to the Cassie–Baxter state.

It has been extensively studied that the hydrophilicity of the nanotubes after anodic oxidation is mainly due to the siphoning effect of the capillary [41], and the hydrophilic hydroxyl group on the surface [42]. In the process of fluorination, these hydroxyl groups were also ‘anchors’ of the chemical binding of FAS, which enhanced the binding area and the bonding strength. The siphon effect of the capillary and the microstructures also increased the contact area of FAS in the fluorination process and enhanced the degree of reaction. Therefore, the improvement of the hydrophobicity of the micro/nano patterned surface was extremely obvious after fluorination, which achieved superhydrophobicity.

## 4. Conclusions

Superhydrophobic surfaces were successfully constructed on Ti-6Al-4V, and the surface wettability was investigated. Micro/nano hybrid structures, including regular microgrooves and nanotube arrays, were fabricated via micro-milling anodic oxidation. Fluorination was used to self-assemble a monolayer on the micro/nano-engineered surface. The microgrooves surface was hydrophobic, because the trapped air in the gaps formed a layer of air cushion, making the surface show a Cassie–Baxter state. After anodic oxidation, the surface turned to hydrophilic, mainly due to the existence of the nanotubes. After fluorination, the surface changed to superhydrophobic with the CA of 153.7° and the contact angle hysteresis of 2.1°. This could be attributed to the synergistic effect of physical morphology and chemical modification. The main reason was that the fluorosilane monolayer formed on the surface reduced the surface free energy. The proposed method for preparing a superhydrophobic titanium surface was considered to have broad application prospects in the field of biomedical materials. The future work will focus on the corrosion resistance and anticoagulant properties of the modified materials.

## Figures and Tables

**Table 1:** Cutting parameters in the micro-milling process.

Spindle Speed (r/min)	Feed Speed (mm/min)	Depth of Cut (μm)
30,000	100	8
